# Comparison of Precision and Accuracy of Five Methods to Analyse Total Score Data

**DOI:** 10.1208/s12248-020-00546-w

**Published:** 2020-12-17

**Authors:** Gustaf J. Wellhagen, Mats O. Karlsson, Maria C. Kjellsson

**Affiliations:** Pharmacometrics Research Group, Department of Pharmacy, Uppsala University, Box 580, 751 23 Uppsala, Sweden

**Keywords:** composite scale data, total score data, bounded integer model, mixed models for repeated measures, IRT-informed total score analysis

## Abstract

Total score (TS) data is generated from composite scales consisting of several questions/items, such as the Movement Disorder Society-Unified Parkinson’s Disease Rating Scale (MDS-UPDRS). The analysis method that most fully uses the information gathered is item response theory (IRT) models, but these are complex and require item-level data which may not be available. Therefore, the TS is commonly analysed with standard continuous variable (CV) models, which do not respect the bounded nature of data. Bounded integer (BI) models do respect the data nature but are not as extensively researched. Mixed models for repeated measures (MMRM) are an alternative that requires few assumptions and handles dropout without bias. If an IRT model exists, the expected mean and standard deviation of TS can be computed through IRT-informed functions—which allows CV and BI models to estimate parameters on the IRT scale. The fit, performance on external data and parameter precision (when applicable) of CV, BI and MMRM to analyse simulated TS data from the MDS-UPDRS motor subscale are investigated in this work. All models provided accurate predictions and residuals without trends, but the fit of CV and BI models was improved by IRT-informed functions. The IRT-informed BI model had more precise parameter estimates than the IRT-informed CV model. The IRT-informed models also had the best performance on external data, while the MMRM model was worst. In conclusion, (1) IRT-informed functions improve TS analyses and (2) IRT-informed BI models had more precise IRT parameter estimates than IRT-informed CV models.

**Supplementary Information:**

The online version contains supplementary material available at 10.1208/s12248-020-00546-w.

## BACKGROUND

Composite scale data is made up of many questions/items with categorical responses that can be summed up through an algorithm into a total score (TS), which is discrete and bounded. Parkinson’s disease is a therapeutic area where no reliable biomarker exists to monitor disease progression and treatment efficacy. Instead, composite scales designed for diagnosis are used for these purposes.

Item response theory (IRT) models make use of item-level information; therefore, well-constructed IRT models are considered the most informative way of analysing such data. They map the disease severity to one or several latent variable(s) (Ψ). However, they are complex to develop, may be difficult to estimate due to a large number of parameters, may take long time to run and sometimes the item-level data is unavailable. The modeller can then turn to alternative models: continuous variable (CV), bounded integer (BI) (1) or less mechanistic models such as mixed models for repeated measures (MMRM).

Different models have different strengths and weaknesses; CV models are commonplace and easy to implement, but they do not respect the scale boundaries nor the discrete nature of the TS data; BI models respect the boundaries and data nature by operating on a latent variable scale (Z) but have not been used as extensively; MMRM is an appropriate alternative to analysis of (co)variance (ANOVA/ANCOVA) in the case of missing data (2–4), where few assumptions need to be made about the response, but requires many parameters to be estimated and the models do not lend themselves to extrapolation. Beta regression (5), an alternative to standard Gaussian CV models, is sometimes used for bounded outcomes (6), which allows for flexible distributions (J- or U-shaped) (7–12). However, this requires a transformation of the data, and besides, the transformation is onto the open interval (0,1) so that the boundaries are not included, which is typically achieved through *Y*^∗^ = (*Y* ∙ (*n* − 1) + 0.5)/*n*, where *n* is the sample size (5). The choice of transformation or correction factor has a large impact on the data at or close to the boundaries, and beta regression has been shown to be statistically non-rigorous (13) and behaves poorly at the boundaries (14). Logit transformation (15) is another option to constrain an outcome to (0,1), which also faces issues at the boundary since these are only asymptotes.

If an IRT model exists for the scale in question, IRT-informed functions of disease progression and standard deviation (SD) can be computed through the item characteristic curves. With these link functions, the goodness-of-fit of CV and BI models for TS analyses can be improved, the latent variable parameters for disease severity of an IRT model can be captured and the relative information of different model types can be compared (Wellhagen GJ, Ueckert S, Kjellsson MC, Karlsson MO. An item response theory-informed strategy to model total score data from composite scales. Forthcoming 2020).

Comparisons of MMRM and CV models have been performed on TS data within the field of Alzheimer’s disease (16,17). It was found that MMRM models are often overparameterised but provide tighter confidence intervals around treatment effects. Since treatment effects are often the primary interest in the late-stage drug development, this is an appealing option to get more certain predictions of drug effect sizes.

In this work, we illustrate the strengths and weaknesses of CV, BI and MMRM models to analyse TS data in a phase 3 clinical trial setting in Parkinson’s disease via simulations from an IRT model. The IRT-informed functions are also evaluated in CV and BI models to improve fit, increase precision and reduce bias of IRT parameters. Also, the precision and bias in the drug effect at end-of-treatment is investigated.

## METHODS

### Simulation Model

A previously published IRT model (18) was used to simulate MDS-UPDRS motor data during 42 months, across 10 visits. A Weibull dropout model was added, see Eqs. (1-2):

1-2 $$ \left\{\begin{array}{c}\rho ={\theta}_1{e}^{-{\theta}_2\frac{t}{12}}\\ {}P(t)=1-{e}^{-{\left(\ln (2)\frac{t}{\rho}\right)}^{\theta_3}}\end{array}\right. $$

where *ρ* is the scale factor, θ_1_ is the baseline mean time to dropout (120 months), θ_2_ is the hazard ratio for the time in the study (0.03, i.e. ~ 3% per year), θ_3_ is the shape factor (set to 2), *t* is time in months and *P*(*t*) is then the resulting probability of dropping out at time *t*. The baseline hazard was also associated with an inter-individual variability (IIV) (proportional with variance: *ω*^2^ = 0.25). The resulting dropout rate (~ 15%) was similar to previously reported in studies of at least 10 weeks (19).

The disease progression was assumed to be linear on the latent variable scale, as was reported in the published IRT model (18).

### Simulation Scenarios

Four different populations were simulated:

1. Relatively healthy (*Ψ*_baseline_ = 0) with slow disease progression (slope = 0.3/year)
2. Relatively healthy with fast disease progression (slope = 0.6/year)
3. Relatively ill (*Ψ*_baseline_ = 1.5) with slow disease progression (slope = 0.3/year)
4. Relatively ill with fast disease progression (slope = 0.6/year)

For each population, three different kinds of drug effects were implemented *vs.* placebo:

- A disease-modifying effect of 30% reduction of the slope
- A symptomatic effect, e.g. offset, on the latent variable with a reduction of 0.315 and 0.63, for patients with slow and fast progression, respectively
- A combination of (a) and (b) with 15% slope reduction and a reduction of 0.1575 and 0.315, for patients with slow and fast progression, respectively

The baseline and slope had additive IIVs with *ω*^2^ = 0.5 and *ω*^2^ = 0.025 respectively, while the drug effects all had proportional IIVs with *ω*^2^ = 0.05. All drug effects were titrated such that they would result in the same absolute difference to placebo at month 42, independent of the drug effect being disease-modifying or symptomatic and patients having a high or low baseline: a difference of 0.315 or 0.63 for patients with slow or fast disease progression, respectively. Drug effects came into act immediately post-baseline. For each scenario (4 × 3 = 12), 100 simulations were run, totalling 1200 studies.

A validation data set containing 1000 individuals (1:1 design) at 10 occasions each (with dropout) was also simulated for each simulation scenario.

### Titration of Study Size

The power was titrated to be the same in all studies. In each of the 12 simulation scenarios, the number of individuals needed per treatment group to identify a drug effect with 80% power at a 5% significance level at 42 months was calculated and rounded up to the nearest 5. A 1:1 parallel design placebo-controlled trial with *n*/arm set to the titrated value was then analysed for each simulation number.

### Estimation Models

The simulated data were analysed once with each of five different models: (1) standard CV (S-CV), (2) IRT-informed CV (I-CV), (3) standard BI (S-BI), (4) IRT-informed BI (I-BI) and (5) MMRM with 1st-order autoregressive residual correlation model (AR1). The definitions for all nonlinear mixed-effects (NLME) models are the same as in (Wellhagen GJ, Ueckert S, Kjellsson MC, Karlsson MO. An item response theory-informed strategy to model total score data from composite scales. Forthcoming 2020), while the MMRM model is only described here.

#### Continuous Variable Models

In the standard CV model (S-CV), the observation *j* for subject *i* at time *t*_*ij*_ is described through:

$$ {\displaystyle \begin{array}{l}{Y}_{ij}=f\left(\theta, {\eta}_i,{t}_{ij},{X}_i\right)+{\varepsilon}_{ij}\\ {}\kern2.5em {\eta}_i\sim N\left(0,{\omega}^2\right)\\ {}\kern2.5em {\varepsilon}_{ij}\sim N\left(0,{\sigma}^2\right)\end{array}} $$

where *θ* is the fixed effect parameters, *η*_*i*_ is the random effects of the inter-individual, *X*_*i*_ is the covariates, *ε*_*ij*_ is the residual unexplained variability (RUV), *ω*^2^ is the variance of the IIV and σ^2^ the variance of the RUV.

The fully IRT-informed CV model (I-CV) is expressed as:

$$ {\displaystyle \begin{array}{c}\begin{array}{c}{\Psi}_{ij}=h\left(\theta, {\eta}_i,{t}_{ij},{X}_i\right)\\ {}{Y}_{ij}={pn}_1\left({\Psi}_{ij}\right)+{\varepsilon}_{ij}\cdotp {pn}_2\left({\Psi}_{ij}\right)\end{array}\\ {}{\eta}_i\sim N\left(0,{\omega}^2\right)\\ {}{\varepsilon}_{ij}\sim N\left(0,1\right)\end{array}} $$

where *Ψ*_*ij*_ is a latent variable described by the nonlinear function *h*(·) and *pn*_1_ as well as *pn*_2_ are predetermined polynomials (Wellhagen GJ, Ueckert S, Kjellsson MC, Karlsson MO. An item response theory-informed strategy to model total score data from composite scales. Forthcoming 2020). The other variables maintain their definition from above.

#### Bounded Integer Models

The standard BI model (S-BI) is a discrete data model, where the probability of an individual *i* to have the score *k* at time *t*_*ij*_ is:

$$ {\displaystyle \begin{array}{c}P\left({Y}_{ij}=k\right)=\phi \left(\frac{Z_{\frac{k}{n}}-f\left(\uptheta, {\upeta}_i,{t}_{ij},{X}_i\right)\ }{g\left(\upsigma, {\upeta}_i,{t}_{ij},{X}_i\right)\ }\right)-\phi \left(\frac{Z_{\frac{k-1}{n}}-f\left(\uptheta, {\upeta}_i,{t}_{ij},{X}_i\right)\ }{g\left(\upsigma, {\upeta}_i,{t}_{ij},{X}_i\right)\ }\right)\\ {}{\eta}_i\sim N\left(0,{\omega}^2\right)\end{array}} $$

where *ϕ* is the cumulative distribution function for the standard normal distribution, *Z*_k/n_ and *Z*_(k-1)/n_ are the cut points between categories *k* and *k*-1 defined through the probit function for an *n*-category scale, *f*(·) is the function for the mean and *g*(·) the function for the variance on the probit scale. For all BI models, the special cases for the first and last categories (*k = 1, k = n*) apply:

$$ P\left({Y}_{ij}=1\right)=\phi \left(\frac{Z_{\frac{1}{n}}-f\left(\uptheta, {\upeta}_i,{t}_{ij},{X}_i\right)\ }{g\left(\upsigma, {\upeta}_i,{t}_{ij},{X}_i\right)\ }\right) $$

$$ P\left({Y}_{ij}=n\right)=1-\phi \left(\frac{Z_{\frac{n-1}{n}}-f\left(\uptheta, {\upeta}_i,{t}_{ij},{X}_i\right)\ }{g\left(\upsigma, {\upeta}_i,{t}_{ij},{X}_i\right)\ }\right) $$

The fully IRT-informed BI model (I-BI) is expressed as:

$$ {\displaystyle \begin{array}{c}{\Psi}_{ij}=h\left(\varTheta, {\eta}_i,{t}_{ij},{X}_i\right)\\ {}P\left({Y}_{ij}=k\right)=\phi \left(\frac{Z_{\frac{k}{n}}-{pn}_3\left({\Psi}_{ij}\right)\ }{pn_4\left({\varPsi}_{ij}\right)\ }\right)-\phi \left(\frac{Z_{\frac{k-1}{n}}-{pn}_3\left({\Psi}_{ij}\right)}{pn_4\left({\varPsi}_{ij}\right)\ }\right)\\ {}{\eta}_i\sim N\left(0,{\omega}^2\right)\end{array}} $$

where *pn*_3_ as well as *pn*_4_ are predetermined polynomials (distinct from *pn*_1_ and *pn*_2_).

#### Mixed Models for Repeated Measures

The MMRM model is defined as:

$$ {\displaystyle \begin{array}{c}{Y}_{ij m}={\theta}_{jm}+{\eta}_i+{\varepsilon}_{ij}\\ {}{\eta}_i\sim N\left(0,{\omega}^2\right)\\ {}{\varepsilon}_{ij}\sim N\left(0,{\sigma_j}^2\right)\end{array}} $$

where *Y*_*ijm*_ is the response of individual *i* at time *j* and dose arm *m*, *θ*_*jm*_ the fixed effect, *η*_*i*_ the random effect of the inter-individual variability (IIV), *ω*^2^ the variance of the IIV, *ε*_*ij*_ the residual unexplained variability (RUV) and *σ*_*j*_^2^ the variance of the RUV. A 1st-order residual correlation was assumed. Two alternative models were tested: either the variance was allowed to vary between the dose arms (*σ*_*m*_^2^, 2 parameters) or between each visit (*σ*_*j*_^2^, 10 parameters).

### Evaluation Metrics

Precision and accuracy of estimated parameters were investigated for the two IRT-informed models (I-CV and I-BI), where the parameters were expressed on the same scale as the IRT model, i.e. on the latent variable Ψ. The precision and accuracy were illustrated by the distribution of the parameter estimates.

Model fit was evaluated through Akaike information criterion (AIC), computed from Objective Function Value (OFV) as in Eq. (3):

3 $$ \mathrm{AIC}=\mathrm{OFV}+2p $$

where *p* is the number of parameters (including IIVs) estimated in the model. The largest model was the MMRM with variance per time point, while the smallest were the I-CV and I-BI since no SD was estimated then. As an example, given a symptomatic drug effect, the S-CV and S-BI models had a total of 8 parameters, the I-CV and I-BI had 6, while the MMRM models had 26 and 34, respectively. The AIC for the S-CV model was used as a reference to compute relative AIC for all other models. The performance on external data of all estimation models, with the final parameter estimates fixed, was also evaluated in the validation data set—via relative OFV since no parameters were estimated.

The goodness-of-fit was also assessed by assessing the proportion of observations, with a residual outside ± 2 SD. Under a standardised residual following *N*(0,1), this number should be 5%. Different measurements of residuals were used for different models due to the nature of the models. For the CV and MMRM models (continuous models), the conditional weighted residual (CWRES) (20) was used and for the BI models (likelihood models), the Pearson individual weighted residual (PIWRES) was used (21). PIWRES should be a standardised residual (*N*(0,1)).

Lastly, the precision and bias of the predicted drug effect at the end-of-study were evaluated with the final parameter estimates; the placebo-corrected prediction of TS (∆TS) was simulated for each scenario with 500 individuals in a crossover design.

### Software

The CV and BI models were evaluated through nonlinear mixed-effects modelling with NONMEM version 7.4 (ICON Development Solutions, Ellicott City, MD), executed through PsN version 4.9 (22,23). The Laplacian estimation method with η-ε interaction was used for all the CV and MMRM models, while BI models were estimated with stochastic approximation and expectation maximisation (SAEM). Importance sampling with an expectation step was added after the estimation step to generate comparable OFVs for all models. Graphics were made with R version 3.6.2 (24) and tidyverse (25). The piraid (26) package was used to create the IRT-informed functions, including the predetermined polynomials (pn_1_-pn_4_) and associated NONMEM control streams.

## RESULTS

The sample size, calibrated to ca 80% power, varied from 25 to 80 subjects/arm depending on the simulation scenario. The lowest sample size was associated with a relatively healthy population with fast disease progression and the highest sample size was needed for the relatively ill population with slow disease progression. In Supplemental Table SI, sample sizes for all the scenarios are tabulated.

For the two MMRM models assessed, a different variance per arm or per time point, the average AIC was always lower for the larger, with variance per time point, model (results not shown). The MMRM models were also evaluated in R (results not shown), where an additional model, with unconstrained residual correlation matrix, was tested. This model did however not improve the fit compared to the AR1 model. Hence, the MMRM model with AR1 and variance per time was chosen for further comparison.

**Fig. 1:**
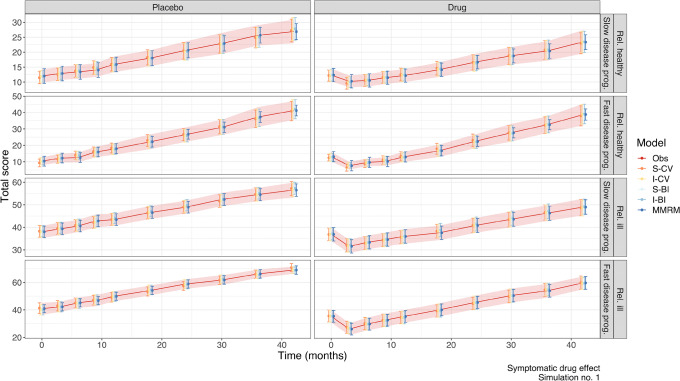
Average and 95% prediction interval (PI) of observations and predictions at each time point for all models, following a symptomatic drug effect for simulation number 1 of 100, stratified by population. The solid line represents the average of the observations and the shaded area represents the PI of the observations. Points represent the average of the predictions for each model and error bars represent the PI of the predictions for each model, with different colours. IRT, item response theory; I-BI, IRT-informed bounded integer model; I-CV, IRT-informed continuous variable model; S-BI, standard bounded integer model; S-CV, standard continuous variable model; MMRM, mixed model for repeated measures

In Fig. 1, the averages of the observed and predicted total score for all models are shown for one simulation to exemplify: a symptomatic drug effect. There was no sign of bias and the precision was similar for all models. Similar plots with examples for the other drug effects are shown in Supplemental Fig. S1 and Supplemental Fig. S2.

**Fig. 2:**
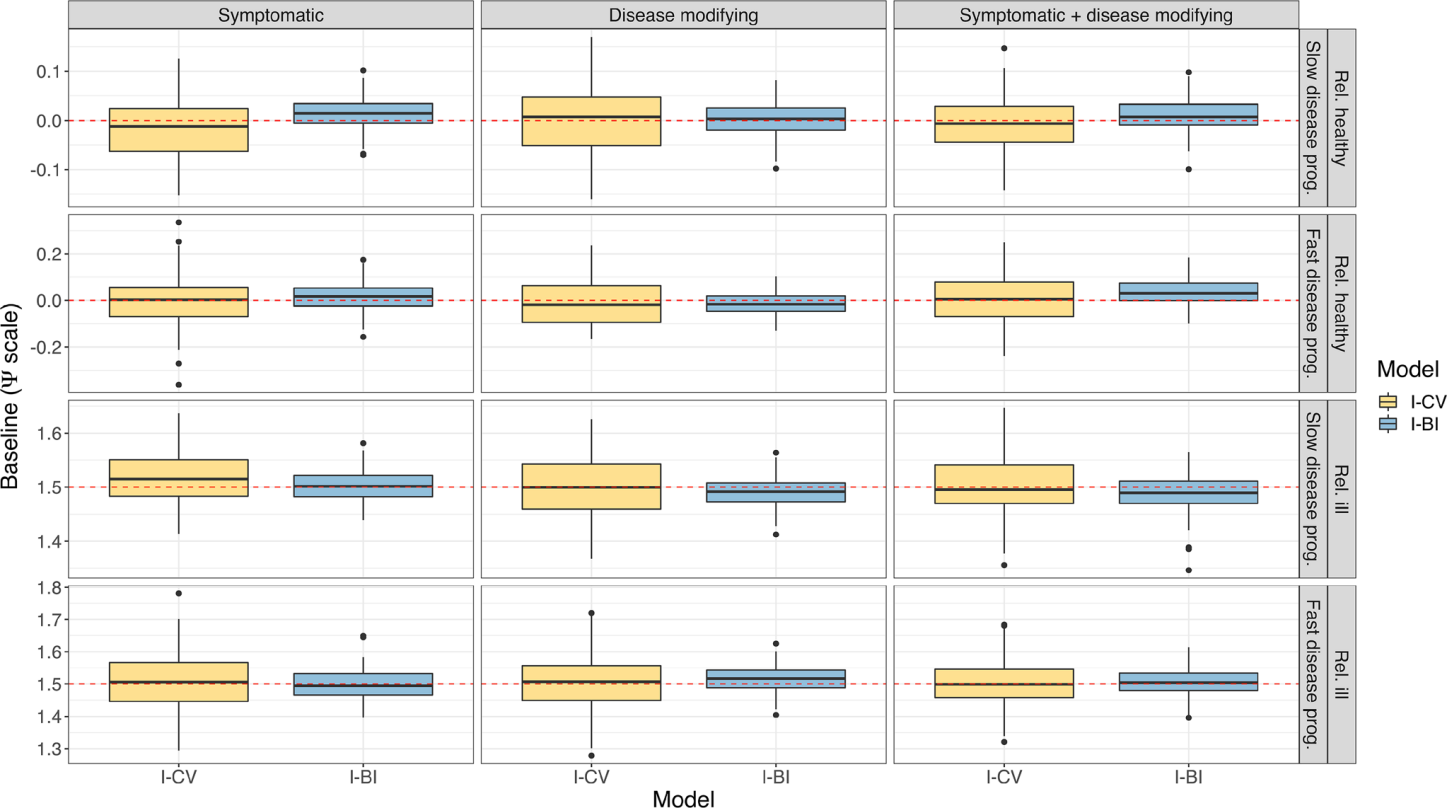
Distribution of the baseline parameter in IRT-informed models, stratified by drug effect and population. The dashed red line indicates the true parameter value. IRT, item response theory; I-CV, IRT-informed continuous variable model; I-BI, IRT-informed bounded integer model

In Fig. 2 and Supplemental Fig. S3, the distributions of the baseline and slope parameters in the IRT-informed models are shown. Neither of the models showed a strong bias in these parameter estimates; however, the I-BI model has a few cases of overprediction of the slope—when simultaneously showing overpredicted disease-modifying drug effect parameter (Supplemental Fig. S6). The I-BI model had more precise estimates of the baseline parameters than the I-CV model. The relative bias of the IIV for these parameters was comparable, shown in Supplemental Fig. S4 and Supplemental Fig. S5.

**Fig. 3:**
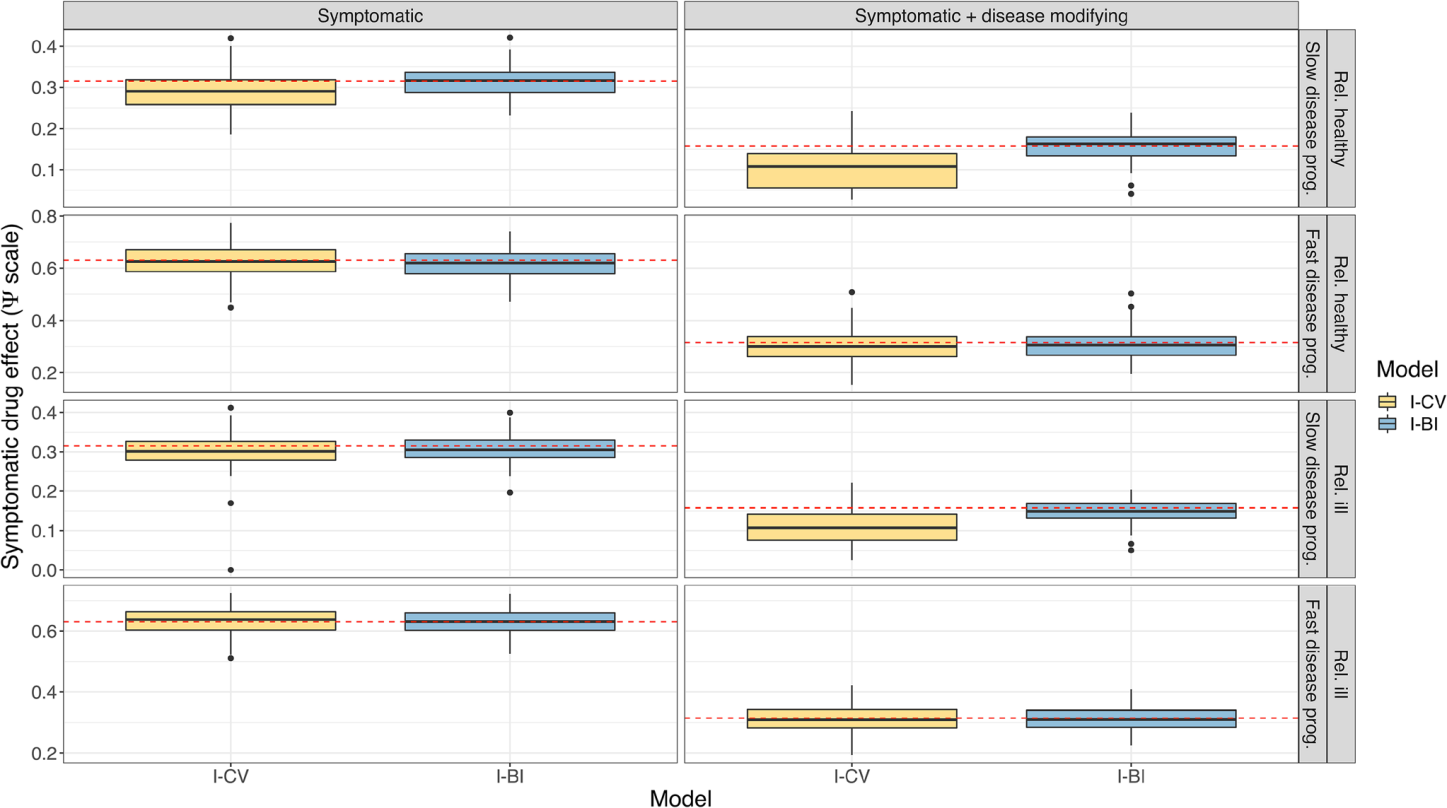
Distribution of the symptomatic drug effect parameter in IRT-informed models, stratified by drug effect and population. The dashed red line indicates the true parameter value. IRT, item response theory; I-CV, IRT-informed continuous variable model; I-BI, IRT-informed bounded integer model

In Fig. 3 and Supplemental Fig. 6, the distributions of the symptomatic and disease-modifying drug effect parameters in the IRT-informed models are shown. The precision of the estimates from the IRT-informed models was similar, but the I-CV model tended to underpredict the symptomatic drug effect parameter when the disease progression was slow, with a more pronounced bias in the combined drug effect. As seen in Supplemental Fig. S7 and Supplemental Fig. S8, both models showed signs of positive bias in estimating the variance of IIV; however, the I-BI model gave considerably more precise estimates. The underprediction may also be a result of these IIVs being implemented lognormally, which means that the median is lower than the mean.

**Fig. 4:**
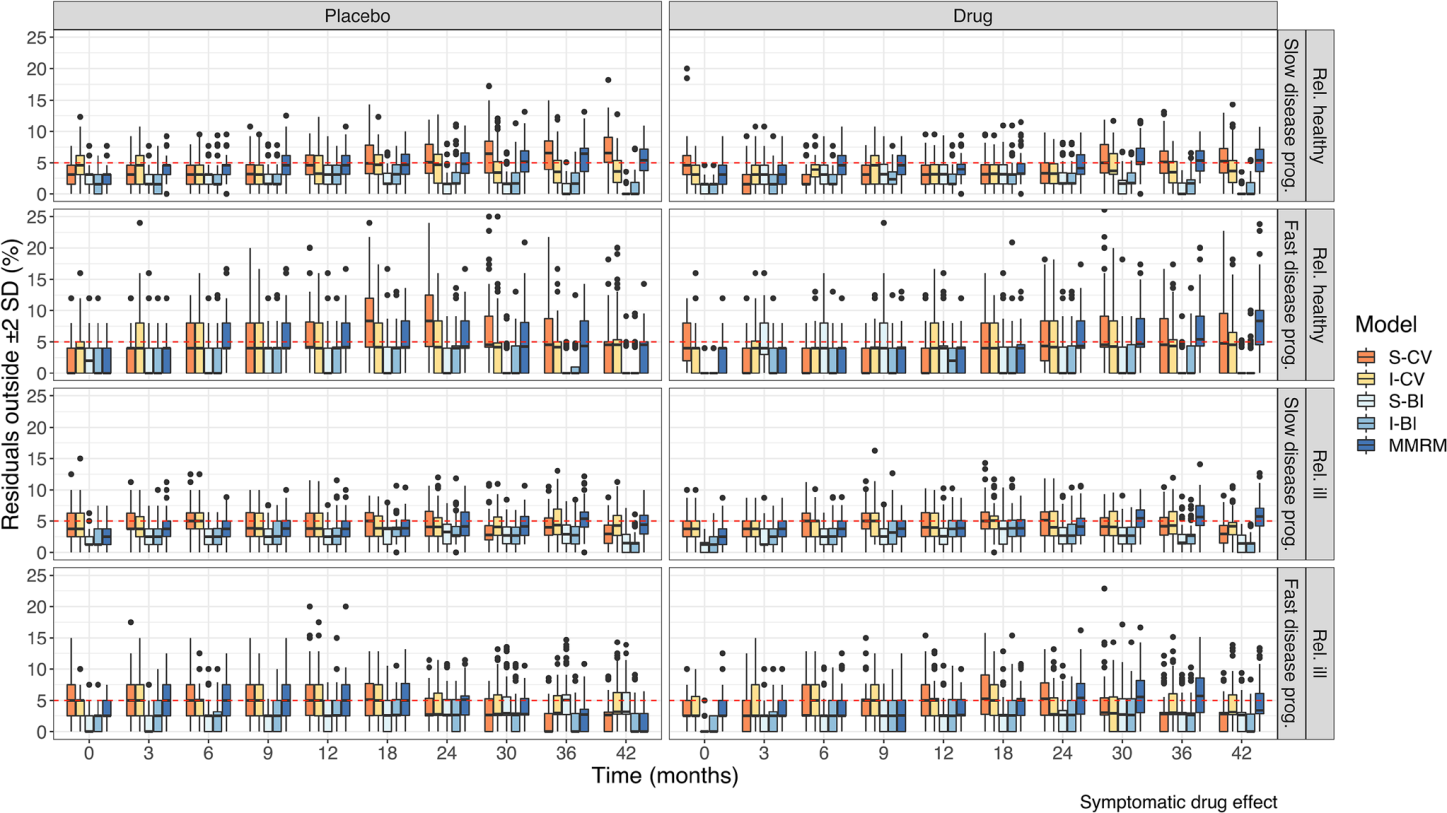
Residual diagnostic showing the percent residuals outside ± 2 standard deviations for all models under a symptomatic drug effect, stratified by population. Note that the y-axis has been cut for visibility. CWRES, conditional weighted residual; IRT, item response theory; I-BI, IRT-informed bounded integer model; I-CV, IRT-informed continuous variable model; MMRM, mixed model for repeated measures; PIWRES, Pearson individual weighted residual; S-BI, standard bounded integer model; S-CV, standard continuous variable model

In Fig. 4, the residual diagnostic for all models across all visits is shown for one example: a symptomatic drug effect. It can be seen that the BI models typically show less than 5% residuals outside 2 standard deviations, while the CV and MMRM models target this number. There is no strong temporal trend, but there are more outliers at the end of the study. Similar plots for the other drug effects are shown in Supplemental Fig. S9 and Supplemental Fig. S10.

**Fig. 5:**
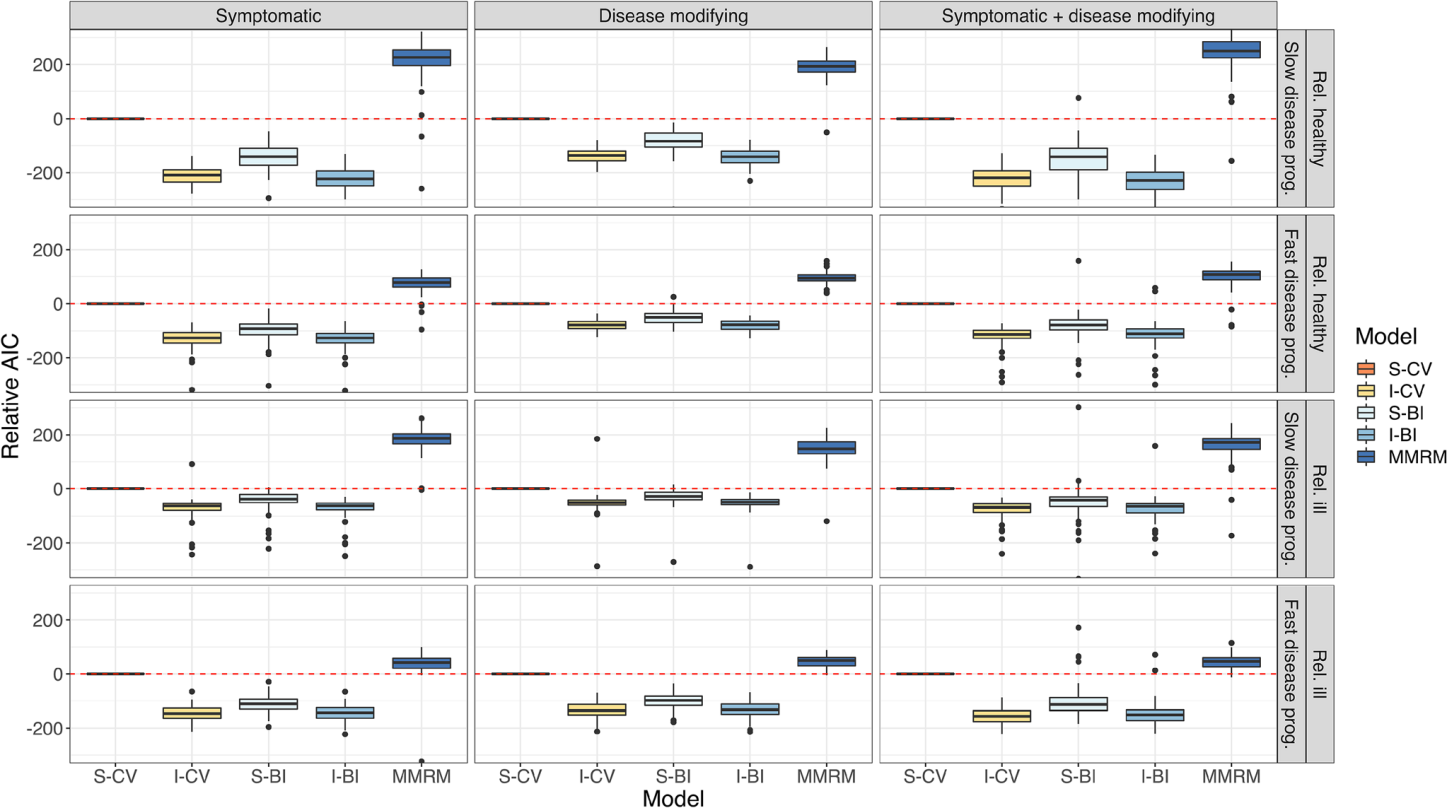
Relative AIC of the investigated models, stratified by drug effect and population. The standard continuous variable (S-CV) model is used as the reference model. Note that the y-axis has been cut for visibility. IRT, item response theory; I-BI, IRT-informed bounded integer model; I-CV, IRT-informed continuous variable model; S-BI, standard bounded integer model; MMRM, mixed model for repeated measures

**Fig. 6:**
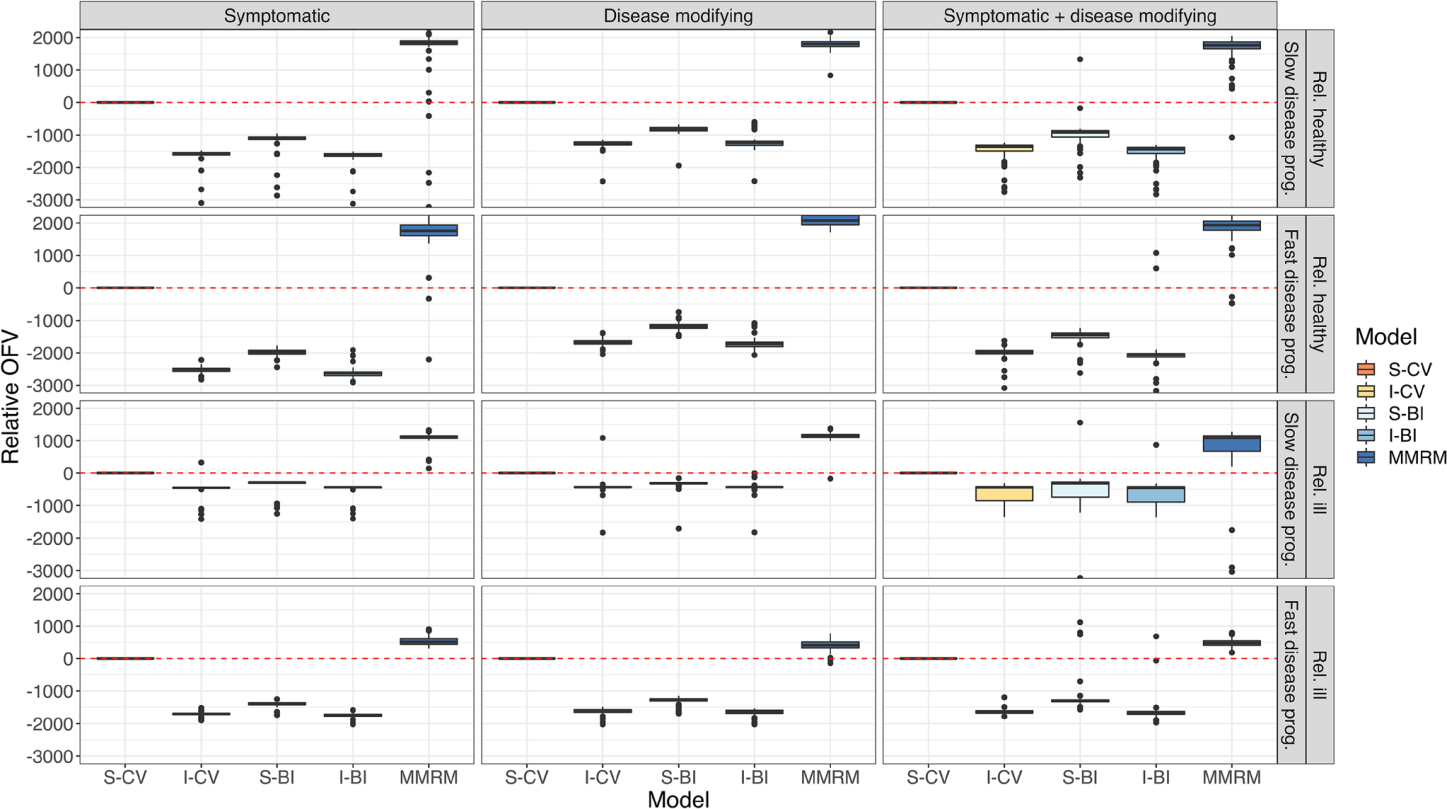
Relative OFV of the investigated models on the validation data set, stratified by drug effect and population. The standard continuous variable (S-CV) model is used as the reference model. Note that the y-axis has been cut for visibility. IRT, item response theory; I-BI, IRT-informed bounded integer model; I-CV, IRT-informed continuous variable model; S-BI, standard bounded integer model; MMRM, mixed model for repeated measures

In Fig. 5, the AIC of all models relative to the S-CV model is shown. The IRT-informed models (I-CV and I-BI) were superior to the standard models and on par with each other. The S-BI offered a better fit than the S-CV model as judged by the relative AIC. The MMRM model had the poorest fit. In Fig. 6, the relative OFV of all models (again compared to the S-CV model) to external data with the final estimates is shown. The same trends were visible there: IRT-informed models had the best fit and MMRM the worst.

**Fig. 7:**
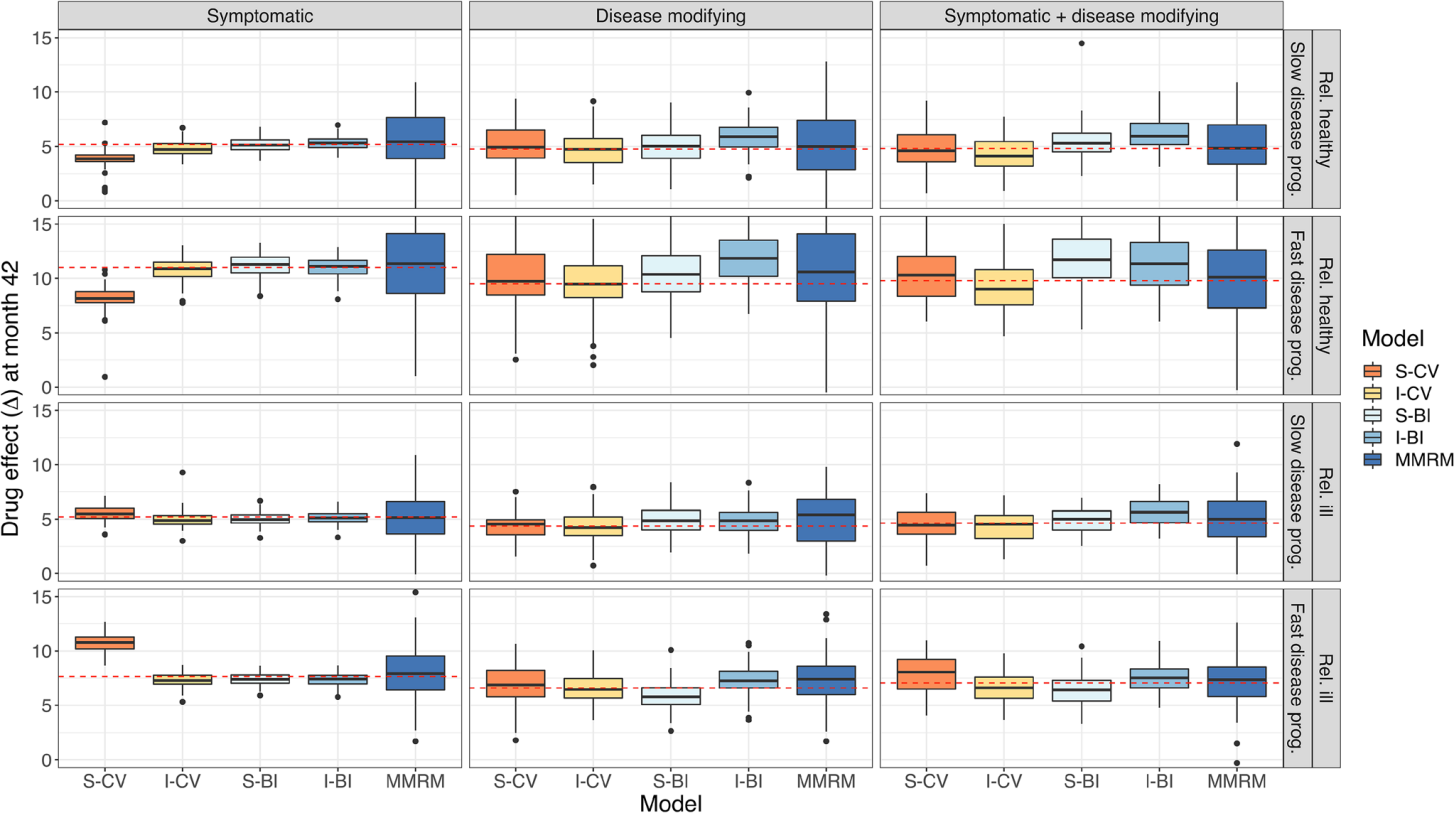
Predicted and true difference between drug and placebo at end-of-study, stratified by drug effect and population. The boxes represent the distribution of differences across 100 predictions. The dashed red line indicates the true, simulated, average difference in 100 simulations. Note that the y-axis has been cut for visibility. IRT, item response theory; I-BI, IRT-informed bounded integer model; I-CV, IRT-informed continuous variable model; S-BI, standard bounded integer model; S-CV, standard continuous variable model; MMRM, mixed model for repeated measures

In Fig. 7, the predicted and true difference from placebo at end-of-study is shown. Mostly, the predictions were close to the true values. The MMRM model always had unbiased predictions, but they were the most imprecise of all, while the S-CV had the most biased predictions. The I-CV model, however, showed no signs of bias. The S-BI and I-BI had a few cases of small bias. It should be noted that the BI models are the only models that predict real life-like data: as integer values.

## DISCUSSION

All the investigated models provided similar predictions, but the possibility to use IRT-informed functions provided improvements in both goodness-of-fit (AIC), due to a better description of the disease progression and SD, as well as performance on external data. In addition, as the IRT-informed functions transform the estimates of the model to the IRT-disease progression scale, parameter estimates can be compared between different model types. The I-CV model was better than the I-BI model at predicting the size of the drug effect without bias, while their respective precision was similar.

For a modeller dealing with TS data, the purpose of the analysis may lead to different model choices. If the goal is to determine a statistical difference between two treatments at the end-of-study, an MMRM model might be the right choice as it is unbiased and structurally simple. However, if anything is to be said about the mechanistic properties of a system or if the model will be used for clinical trial simulations, a full longitudinal model (like CV or BI) should be chosen. The standard BI model is an improvement over the standard CV model, as it respects the bounded data nature and it also allows the simulation of real life-like data. When adding IRT-informed link functions, there was not as clear a difference between I-CV and I-BI, but the simulation properties are still the same. Also, it is then possible to combine information from different model types. This could be valuable when designing new clinical trials. However, the IRT-informed models obviously require that there already exists an IRT model for the scale, which may not always be the case. It should also be a well-constructed IRT model; if it is misspecified, then so will the IRT-informed models be too since the link functions are exact. The pros and cons of the models investigated in this work are summarised in Table I.

**Table I:** The Pros and Cons of Different Methods of Total Score Analysis

Model	Pros	Cons
S-CV	Easy to implement	Does not respect scale boundaries nor data nature, assumes homoscedastic SD
I-CV	Better fit than S-CV, allows to estimate IRT parameters, unbiased predictions	Requires existing IRT model, does not respect data nature
S-BI	Respects data nature and scale boundaries, can simulate real life-like data	Assumes homoscedastic SD (on Z scale)
I-BI	Better fit than S-BI, respects data nature and scale boundaries, allows to estimate IRT parameters, can simulate real life-like data, better IRT parameter precision than I-CV	Requires existing IRT model
MMRM	Unbiased, few mechanistic assumptions needed, robust to model misspecification	Many parameters to estimate, does not respect scale boundaries nor data nature, cannot extrapolate

*BI*, bounded integer; *CV*, continuous variable; *IRT*, item response theory; *I-BI*, IRT-informed BI; *I-CV*, IRT-informed CV; *MMRM*, mixed models for repeated measures; *Z*, latent variable in BI model

Coarsened grid or beta regression models could have been investigated (15,27); coarsened grid models would likely have similar properties to the BI model as they also map the TS to a latent variable. Ordered categorical models could also be applied to composite scale data, but they are parameter heavy and cannot predict data categories not present in the data. They do however respect the boundaries of TS data. In scales with fewer categories one could entertain ordered categorical models, but they are typically not considered for outcomes with more than maximally 10–20 categories. The properties of a categorical MMRM model, which has been described previously (28) but is not routinely used, would also be highly interesting to explore.

The MMRM model provides a robust alternative where no structural parameters except the mean at each time point and the autoregressive parameters need to be estimated. This means that they are robust to model misspecification. MMRM models are inherently unbiased under dropout if the correct (or an unconstrained) correlation structure is used (2,29,30) and were also unbiased in this work—but had the least precise predictions. Also, the fit was not as good as with the other models in this comparison. This is likely because the AR1 structure could be seen as a model misspecification, as it does not offer the same flexibility as the nonlinear models with IIV, especially IIV on SD. Also, since no slope or offset parameter is estimated, it is not possible to directly make inferences about drug effects in terms of these entities. Model averaging across the investigated models, which is a possible extension of this work, should have revealed the misspecification of MMRM models by rendering low weights. The MMRM models offer large flexibility with respect to the time profile. Unconstrained correlation matrices are conventional, while we used an AR1 matrix. However, both AR1 and unconstrained matrix models were evaluated in R and provided a similar fit (results not shown). Notably, the AR1 model was much faster to run in NONMEM. We also implemented an additive random effect in the model, which does not constrain the predictions to be positive. We believe this is the standard implementation that MMRM users would adopt in statistical software since a lognormal distribution as used in the S-CV model might not be easy to implement in most software; however, the exponential model was also evaluated in NONMEM (results not shown) with similar results as the additive random effect.

As residuals are one important way to diagnose model misspecification, the expected properties of residuals should be known. For CV models, these have been studied extensively while the discrete data models, like BI, are not as well documented. In this work, we saw that the PIWRES most often gave less than 5% outliers, defined as > ± 2 SD. This may indicate that the behaviour of the PIWRES metric does not follow that of the standard normal distribution, where ~ 95% of the data should be within ± 2 SD and that perhaps ± 1.5 SD is more reasonable to use with PIWRES, in order to identify outliers or model misspecification. Alternatively, simulation-based residuals such as normalised prediction distribution errors (NPDE) (31) or quantile residuals (32–34) could be considered, which rescale the residuals to a normal (NPDE) or uniform (quantile) distribution.

The dropout model was only affected by time, and not by disease severity, thus following a missing completely at random (MCAR) mechanism. It is possible that patients who experience severe symptoms of parkinsonism would be more likely to drop out; however, no large differences in dropout rates between treatment and placebo groups were observed in the studies that formed the basis for the overall dropout target of 15% (19). The MMRM models are valid under missingness at random (MAR). If missingness was not at random (MNAR), a model for the missingness would need to be implemented in the analysis models to avoid bias and imprecision, which can be done in the more mechanistic models, but not in MMRM. This was however out of the scope of this analysis. The amount of dropout also affects the analysis: more dropouts would mean fewer individuals at the end and hence more imprecise estimates for the MMRM model, while the fully longitudinal models handle this phenomenon better, at the cost of higher shrinkage.

We assumed a direct offset effect that was effective immediately after baseline, a simple model, to clearly illustrate the differences between the analysis models. Since the analysis was based (and titrated to the same power via a *t*-test) on the outcome at 42 months, this should have no impact on the results. Also, the number of simulations could have been greater than the 100 used here; however, it was sufficient for detecting trends in the differences in precision and bias between the investigated models.

## CONCLUSIONS

There are many ways to model TS data, and their respective strengths and weaknesses have been highlighted in this work, along with recommendations for when to choose a certain method. For unbiased statistical tests, MMRM appears well suited, but the other methods were more precise in their predictions. For simulations, NLME models, rather than MMRM, need to be chosen. Standard CV models are easy to implement, but BI models are the only ones that respect the discrete data nature. The IRT-informed models (I-CV and I-BI) models provided the best fit and also the best performance on external data. Furthermore, the IRT link functions allow IRT parameters to be retrieved with high precision and low bias—especially in the I-BI model. This will aid modellers analysing clinical trial data with total scores to choose a fit-for purpose analysis method.

## Supplementary Information

Supplemental Figure S1. Average and 95% prediction interval (PI) of observations and predictions at each time point for all models, following a disease-modifying drug effect for simulation number 1 of 100, stratified by population. The solid line represents the average of the observations and the shaded area represents the PI of the observations. Points represent the average of the predictions for each model and error bars represent the PI of the predictions for each model, with different colours. IRT, item response theory; I-BI, IRT-informed bounded integer model; I-CV, IRT-informed continuous variable model; S-BI, standard bounded integer model; S-CV, standard continuous variable model; MMRM, mixed model for repeated measures. (PDF 70 kb)

Supplemental Figure S2. Average and 95% prediction interval (PI) of observations and predictions at each time point for all models, following a combined symptomatic and disease-modifying drug effect for simulation number 1 of 100, stratified by population. The solid line represents the average of the observations and the shaded area represents the PI of the observations. Points represent the average of the predictions for each model and error bars represent the PI of the predictions for each model, with different colours. IRT, item response theory; I-BI, IRT-informed bounded integer model; I-CV, IRT-informed continuous variable model; S-BI, standard bounded integer model; S-CV, standard continuous variable model; MMRM, mixed model for repeated measures. (PDF 70 kb)

Supplemental Figure S3 Distribution of the slope parameter in IRT-informed models, stratified by drug effect and population. The dashed red line indicates the true parameter value. IRT, item response theory; I-CV, IRT-informed continuous variable model; I-BI, IRT-informed bounded integer model. (PDF 49 kb)

Supplemental Figure S4 Distribution of the IIV of the baseline parameter in IRT-informed models, stratified by drug effect and population. The dashed red line indicates the true parameter value. IRT, item response theory; I-CV, IRT-informed continuous variable model; I-BI, IRT-informed bounded integer model. (PDF 47 kb)

Supplemental Figure S5. Relative bias of the IIV of the slope parameter in IRT-informed models, stratified by drug effect and population. The dashed red line indicates the true parameter value. IRT, item response theory; I-CV, IRT-informed continuous variable model; I-BI, IRT-informed bounded integer model. (PDF 48 kb)

Supplemental Figure S6. Distribution of the disease-modifying drug effect parameter in IRT-informed models, stratified by drug effect and population. The dashed red line indicates the true parameter value. IRT, item response theory; I-CV, IRT-informed continuous variable model; I-BI, IRT-informed bounded integer model. (PDF 555 kb)

Supplemental Figure S7. Distribution of the IIV of the symptomatic drug effect parameter in IRT-informed models, stratified by drug effect and population. The dashed red line indicates the true parameter value. Note that the y axis has been cut for visibility. IRT, item response theory; I-CV, IRT-informed continuous variable model; I-BI, IRT-informed bounded integer model. (PDF 46 kb)

Supplemental Figure S8. Distribution of the IIV of the disease-modifying drug effect parameter in IRT-informed models, stratified by drug effect and population. The dashed red line indicates the true parameter value. Note that the y axis has been cut for visibility. IRT, item response theory; I-CV, IRT-informed continuous variable model; I-BI, IRT-informed bounded integer model. (PDF 47 kb)

Supplemental Figure S9. Residual diagnostic showing the percent residuals outside ±2 standard deviations for all models under a disease-modifying drug effect, stratified by population. Note that the y axis has been cut for visibility. CWRES, conditional weighted residual; IRT, item response theory; I-BI, IRT-informed bounded integer model; I-CV, IRT-informed continuous variable model; MMRM, mixed model for repeated measures; PIWRES, Pearson individual weighted residual; S-BI, standard bounded integer model; S-CV, standard continuous variable model. (PDF 73 kb)

Supplemental Figure S10. Residual diagnostic showing the percent residuals outside ±2 standard deviations for all models under a combined symptomatic and disease-modifying drug effect, stratified by population. Note that the y axis has been cut for visibility. CWRES, conditional weighted residual; IRT, item response theory; I-BI, IRT-informed bounded integer model; I-CV, IRT-informed continuous variable model; MMRM, mixed model for repeated measures; PIWRES, Pearson individual weighted residual; S-BI, standard bounded integer model; S-CV, standard continuous variable model. (PDF 74 kb)

Supplemental Table SI (DOCX 12 kb)
