# Supplementary material for: Comparison of Precision and Accuracy of Five Methods to Analyse Total Score Data
Source: AAPS J. 2020 Dec 17;23(1):9. doi: 10.1208/s12248-020-00546-w (PMC7746559; doi:10.1208/s12248-020-00546-w)
Supplement: Supplementary file 1 — Average and 95% prediction interval (PI) of observations and predictions at each time point for all models, following a disease-modifying drug effect for simulation number 1 of 100, stratified by population. The solid line represents the average of the observations and the shaded area represents the PI of the observations. Points represent the average of the predictions for each model and error bars represent the PI of the predictions for each model, with different colours. IRT, item response theory; I-BI, IRT-informed bounded integer model; I-CV, IRT-informed continuous variable model; S-BI, standard bounded integer model; S-CV, standard continuous variable model; MMRM, mixed model for repeated measures. (PDF 70 kb) [file 12248_2020_546_MOESM1_ESM.pdf]

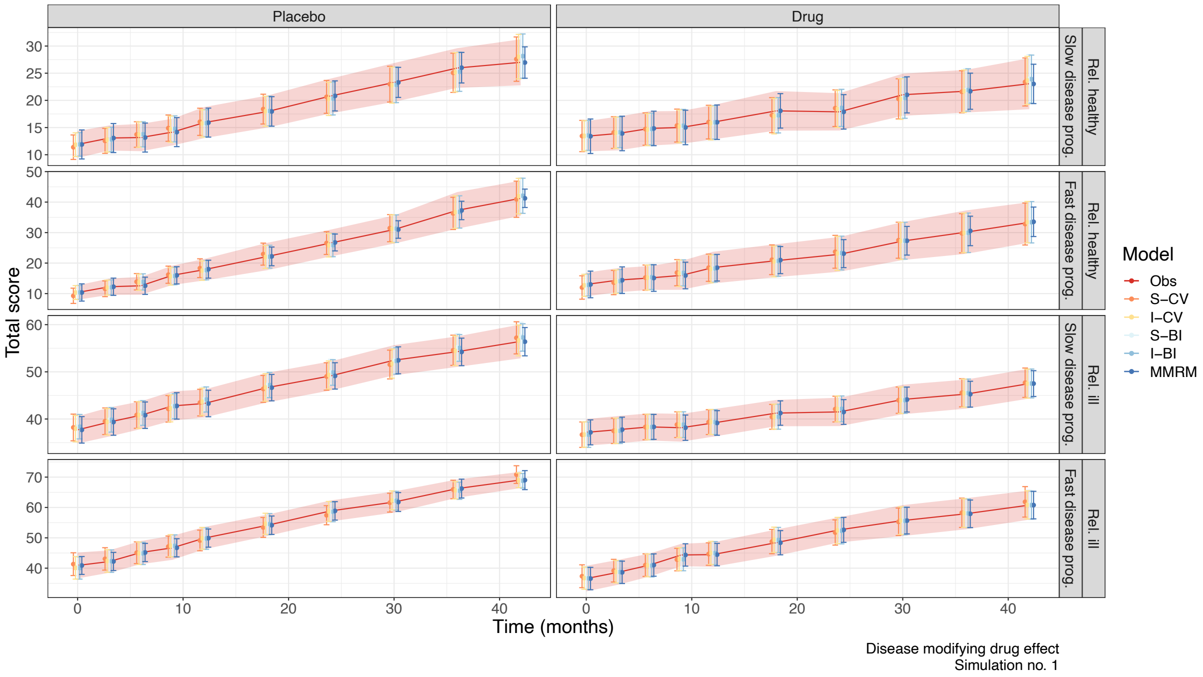

Supplemental Figure S1. Average and 95% prediction interval (PI) of observations and predictions at each time point for all models, following a disease modifying drug effect for simulation number 1 of 100, stratified by population. The solid line represents the average of the observations and the shaded area represents the PI of the observations. Points represent the average of the predictions for each model and error bars represent the PI of the predictions for each model, with different colours. IRT, item response theory; I-BI, IRT-informed bounded integer model; I-CV, IRT-informed continuous variable model; S-BI, standard bounded integer model; S-CV, standard continuous variable model; MMRM, mixed model for repeated measures.
