# Supplementary material for: Comparison of Precision and Accuracy of Five Methods to Analyse Total Score Data
Source: AAPS J. 2020 Dec 17;23(1):9. doi: 10.1208/s12248-020-00546-w (PMC7746559; doi:10.1208/s12248-020-00546-w)
Supplement: Supplementary file 8 — Distribution of the IIV of the disease-modifying drug effect parameter in IRT-informed models, stratified by drug effect and population. The dashed red line indicates the true parameter value. Note that the y axis has been cut for visibility. IRT, item response theory; I-CV, IRT-informed continuous variable model; I-BI, IRT-informed bounded integer model. (PDF 47 kb) [file 12248_2020_546_MOESM8_ESM.pdf]

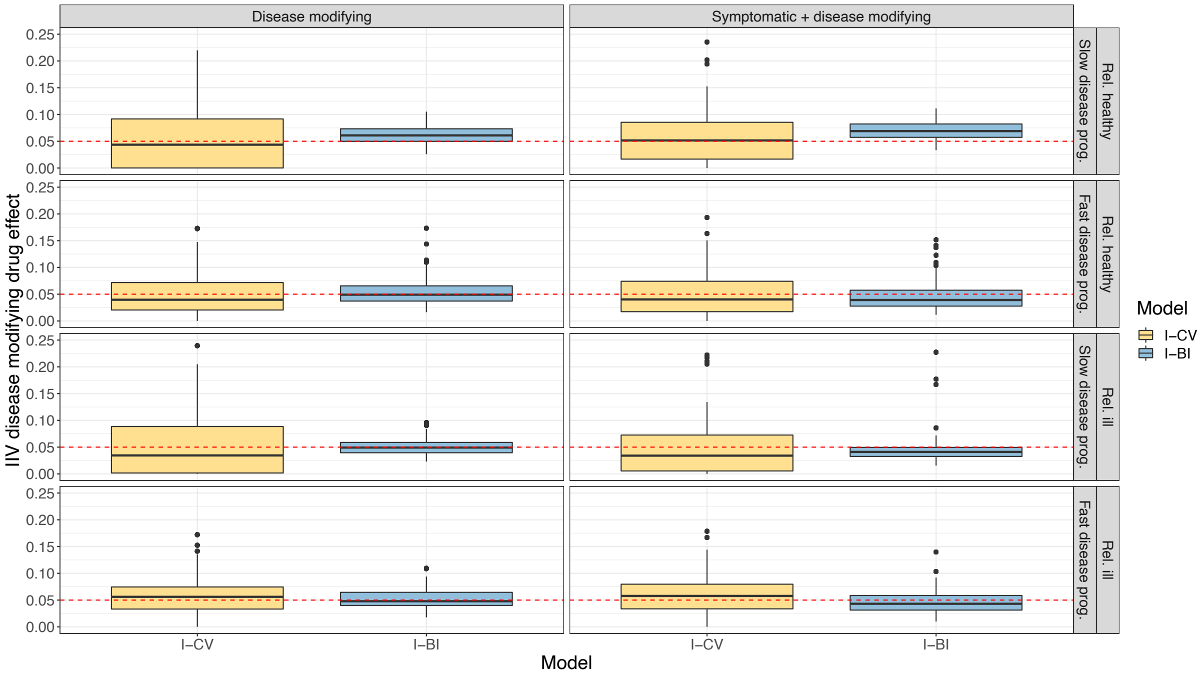

Supplemental Figure S8. Distribution of the IIV of the disease modifying drug effect parameter in IRT-informed models, stratified by drug effect and population. The dashed red line indicates the true parameter value. Note that the y axis has been cut for visibility. IRT, item response theory; I-CV, IRT-informed continuous variable model; I-BI, IRT-informed bounded integer model.
