# Supplementary material for: Comparison of Precision and Accuracy of Five Methods to Analyse Total Score Data
Source: AAPS J. 2020 Dec 17;23(1):9. doi: 10.1208/s12248-020-00546-w (PMC7746559; doi:10.1208/s12248-020-00546-w)
Supplement: Supplementary file 9 — Residual diagnostic showing the percent residuals outside ±2 standard deviations for all models under a disease-modifying drug effect, stratified by population. Note that the y axis has been cut for visibility. CWRES, conditional weighted residual; IRT, item response theory; I-BI, IRT-informed bounded integer model; I-CV, IRT-informed continuous variable model; MMRM, mixed model for repeated measures; PIWRES, Pearson individual weighted residual; S-BI, standard bounded integer model; S-CV, standard continuous variable model. (PDF 73 kb) [file 12248_2020_546_MOESM9_ESM.pdf]

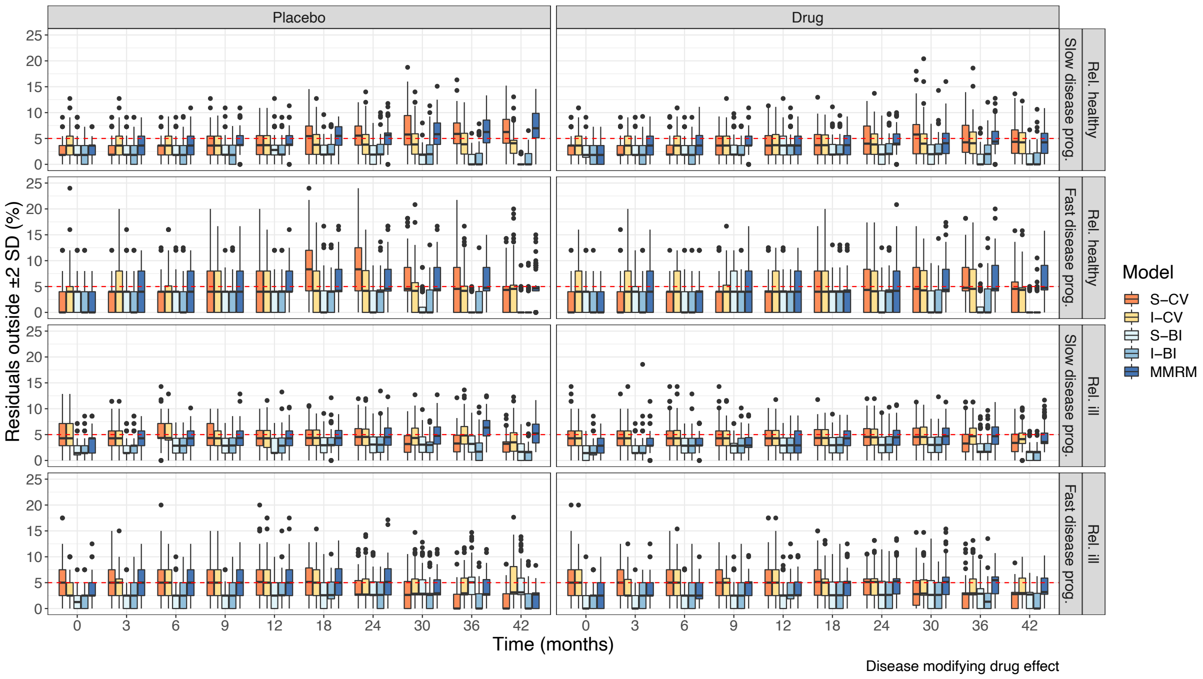

Supplemental Figure S9. Residual diagnostic showing the percent residuals outside ±2 standard deviations for all models under a disease modifying drug effect, stratified by population. Note that the y axis has been cut for visibility. CWRES, conditional weighted residual; IRT, item response theory; I-BI, IRT-informed bounded integer model; I-CV, IRT-informed continuous variable model; MMRM, mixed model for repeated measures; PIWRES, Pearson individual weighted residual; S-BI, standard bounded integer model; S-CV, standard continuous variable model.
