# Supplementary material for: Comparison of Precision and Accuracy of Five Methods to Analyse Total Score Data
Source: AAPS J. 2020 Dec 17;23(1):9. doi: 10.1208/s12248-020-00546-w (PMC7746559; doi:10.1208/s12248-020-00546-w)
Supplement: Supplementary file 11 — (DOCX 12 kb) [file 12248_2020_546_MOESM11_ESM.docx]

Supplemental Table I. Sample sizes required for 80% power at $\alpha=0.05$ for different population and drug effect combinations

| Baseline | Disease progression | Drug effect | | |
| --- | --- | --- | --- | --- |
|  |  | Symptomatic | Disease modifying | Symptomatic + disease modifying |
| Relatively healthy | Slow | 65 | 55 | 75 |
|  | Fast | 25 | 25 | 30 |
| Relatively ill | Slow | 80 | 70 | 80 |
|  | Fast | 40 | 40 | 45 |
